# Initiation of bacteriophage T4 DNA replication and replication fork dynamics: a review in the Virology Journal series on bacteriophage T4 and its relatives

**DOI:** 10.1186/1743-422X-7-358

**Published:** 2010-12-03

**Authors:** Kenneth N Kreuzer, J Rodney Brister

**Affiliations:** 1 Department of Biochemistry, Duke University Medical Center, Durham, NC 27710 USA; 2 National Center for Biotechnology Information, National Library of Medicine, National Institutes of Health, Bethesda, MD 20894 USA

## Abstract

Bacteriophage T4 initiates DNA replication from specialized structures that form in its genome. Immediately after infection, RNA-DNA hybrids (R-loops) occur on (at least some) replication origins, with the annealed RNA serving as a primer for leading-strand synthesis in one direction. As the infection progresses, replication initiation becomes dependent on recombination proteins in a process called recombination-dependent replication (RDR). RDR occurs when the replication machinery is assembled onto D-loop recombination intermediates, and in this case, the invading 3' DNA end is used as a primer for leading strand synthesis. Over the last 15 years, these two modes of T4 DNA replication initiation have been studied *in vivo *using a variety of approaches, including replication of plasmids with segments of the T4 genome, analysis of replication intermediates by two-dimensional gel electrophoresis, and genomic approaches that measure DNA copy number as the infection progresses. In addition, biochemical approaches have reconstituted replication from origin R-loop structures and have clarified some detailed roles of both replication and recombination proteins in the process of RDR and related pathways. We will also discuss the parallels between T4 DNA replication modes and similar events in cellular and eukaryotic organelle DNA replication, and close with some current questions of interest concerning the mechanisms of replication, recombination and repair in phage T4.

## Introduction

Studies during the last 15 years have provided strong evidence that T4 DNA replication initiates from specialized structures, namely R-loops for origin-dependent replication and D-loops for recombination-dependent replication (RDR). The roles of many of the T4 replication and recombination proteins in these processes are now understood in detail, and the transition from origin-dependent replication to RDR has been ascribed to both down-regulation of origin transcripts and activation of the UvsW helicase, which unwinds origin R-loops.

One of the interesting themes that emerged in studies of T4 DNA metabolism is the extensive overlap between different modes of replication initiation and the processes of DNA repair, recombination, and replication fork restart. As discussed in more detail below, the distinction between origin-dependent and recombination-dependent replication is blurred by the involvement of recombination proteins in certain aspects of origin replication. Another example of overlap is the finding that repair of double-strand breaks (DSBs) in phage T4 infections occurs by a mechanism that is very closely related to the process of RDR. The close interconnections between recombination and replication are not unique to phage T4 - it has become obvious that the process of homologous recombination and particular recombination proteins play critical roles in cellular DNA replication and the maintenance of genomic stability [1-4].

### Origin-dependent replication

Most chromosomes that have been studied include defined loci where DNA synthesis is initiated. Such origins of replication have unique physical attributes that contribute to the assembly of processive replisomes, facilitate biochemical transactions by the replisome proteins to initiate DNA synthesis, and serve as key sites for the regulation of replication timing. While the actual determinants of origin activity remain ill defined in many systems, all origins must somehow promote the priming of DNA synthesis. Bacteriophage T4 contains several replication origins that are capable of supporting multiple rounds of DNA synthesis [5,6] and has very well-defined replication proteins [7], making this bacteriophage an ideal model to study origin activation and maintenance.

### Localization of T4 origins throughout the genome

Clear evidence for defined T4 origin sequences began to emerge about 30 years ago when the Kozinski and Mosig groups demonstrated that nascent DNA produced early during infection originated from specific regions within the 169 kb phage genome [8-10]. The race was on, and several groups spent the better part of two decades trying to define the T4 origins of replication. These early efforts brought a battery of techniques to bear, including electron microscopy and tritium labeling of nascent viral DNA, localizing origins to particular regions of the genome. The first direct evidence for the DNA sequence elements that constitute a T4 origin emerged from studies of Kreuzer and Alberts [11,12], who isolated small DNA fragments that were capable of driving autonomous replication of plasmids during a T4 infection. Later approaches using two-dimensional gel electrophoresis confirmed that these two origins, *oriF *and *oriG *[also called *ori(uvsY) *and o*ri(34)*, respectively], were indeed active in the context of the phage genome [13,14]. All told, at least seven putative origins (termed *oriA *through *oriG*) were identified by these various efforts, yet no strong consensus emerged as whether all seven were *bona fide *origins and how the multiple origins were utilized during infection.

**Figure 1:**
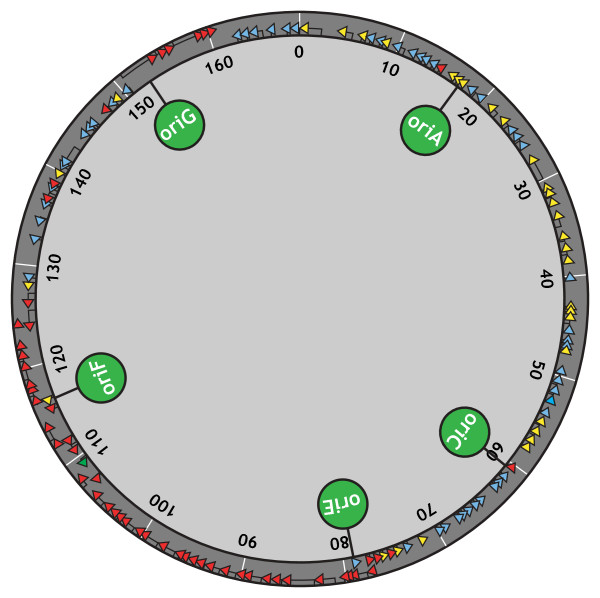
**Location of the T4 origins of replication **. The linear 169 kb T4 genome is circularly permuted and has no defined telomeres, so it is depicted in this diagram as a circle. The positions of major T4 origins are indicated with green lollypops. The positions of major T4 open reading frames (>100 amino acids) are indicated with arrows and are color coded to indicate the timing of transcription: blue, early; yellow, middle; and red, late transcripts [5,19]. Three relevant smaller open reading frames are also included: *soc *near *oriA*; *rI.-1 *near *oriC*; and *repEA *near *oriE*.

Recent work by Brister and Nossal [5,15] has helped to clarify many issues regarding T4 origin usage. Using an array of PCR fragments, they monitored the accumulation of nascent DNA across the entire viral genome over the course of infection, allowing both the origins and breadth of DNA synthesis to be monitored in real time. This whole-genome approach revealed that at least 5 origins of replication are active early during infection, *oriA*, *oriC*, *oriE*, *oriF*, and *oriG *(see Figure 1). Though all of these origins had been independently identified to some extent in previous studies, this was the first observation of concurrent activity from each within a population of infected cells.

There do not appear to be any local sequence motifs shared among all the T4 origins. However, one origin, *oriE*, does include a cluster of evenly spaced, 12-nt direct repeats [16]. Similar "iterons" are also found within syntenic regions of closely related bacteriophage genomes, implying conserved function [17]. Indeed, this arrangement of direct repeats is reminiscent of some plasmid origins, such as the RK6 gamma origin, where replication initiator proteins bind to direct repeats and promote assembly of replisomes [18]. Despite this circumstantial evidence, no association has been established between the T4 iterons and *oriE *replication activity, and to this date their role during T4 infection remains ill defined.

There is some indication that global genome constraints influence the position of T4 origins. Three of the more active T4 origins, *oriE*, *oriF*, and *oriG *are located near chromosomal regions where the template for viral transcription switches from predominately one strand to predominately the complementary strand [5,19] (see Figure 1). These regions of transcriptional divergence coincide with shifts in nucleotide compositional bias (predominance of particular nucleotides on a particular strand), a hallmark of replication origins in other systems [20]. That said, at least two origins (*oriA *and *oriC*) are well outside regions of intrastrand nucleotide skews and transcriptional divergence, so it is not clear what, if any, physical properties of the T4 chromosome contribute to origin location. Moreover, the T4 genome is circularly permuted with no defined telomeres, so the actual position of a given locus relative to the chromosome ends is variable in a population of replicating virus.

The undulating T4 transcription pattern reflects the modular nature of the viral genome. T4 genes are arranged in functionally related clusters, and diversity among T4-related viruses appears to arise through the horizontal transfer of gene clusters [17,21]. The spacing of T4 origins over the length of the viral genome coincides with some of these clusters and may reflect genome mechanics. Most early T4 DNA synthesis originates from regions within the genome that are dominated by late-mode viral transcription [5,19]. This arrangement suggests an intimate relationship between T4 replication and transcription of late genes, like those encoding viral capsid components. It has been known for some time that late-mode transcription is dependent on gp45 clamp protein, which is a component of both the T4 replisome and late-mode transcription complexes (reviewed by Miller *et al*. [22]), but there is also evidence that the amount of replication directly influences the amount of transcription [23] (Brister, unpublished data).

### Molecular mechanism of origin initiation

Though few obvious sequence characteristics are shared between them, all of the T4 origins are thought to facilitate formation of RNA primers used to initiate leading strand DNA synthesis. Most of what is known about the detailed mechanism of T4 replication initiation comes from studies of the two origins (*oriF *and *oriG*) that support autonomous replication of plasmids in T4-infected cells (see above). Origin plasmid replication requires the expected T4-encoded replisome proteins, and like phage genomic DNA replication, is substantially reduced and/or delayed by mutations in the replicative helicase, primase and topoisomerase [24,25].

The DNA sequences required for *oriF *and *oriG *function on recombinant plasmids have been defined by deletion and point mutation studies [26] (Menkens and Kreuzer, unpublished data). A minimal sequence of about 100 bp from each origin was shown to be necessary for autonomous replication, and though there is little homology between *oriF *and *oriG*, both minimal sequences include a middle-mode promoter and an A + T-rich downstream unwinding element (DUE) [26,27]. Middle-mode promoters consist of a binding site for the viral transcription factor MotA in the -30 region, along with a -10 sequence motif that is indistinguishable from the typical *E. coli σ*70 -10 motif [28,29]. Transcripts initiated from the *oriF *MotA-dependent promoter were shown to form persistent R-loops within the DUE region, leaving the non-template strand hypersensitive to ssDNA cleavage. Formation of these R-loops is not dependent on specific sequences and the endogenous DUE can be substituted with heterologous unwinding elements [13,27].

The *oriF *R-loops are very likely processed by viral RNase H to generate free 3'-OH ends that are used to prime leading strand DNA synthesis [13,27]. Furthermore, the presence of an R-loop presumably holds the origin duplex in an open conformation, giving the gp41/61 primosome complex access to the unpaired non-template strand to allow extensive parental DNA unwinding and priming on the lagging strand. Less is known about replication priming at the other T4 origins [30]. Presumably, *oriG *uses the same mechanism as *oriF *[13,27], and there is some evidence that a transcript from a nearby MotA-dependent promoter is used to initiate replication at *oriA *[30]. Yet, MotA mutations do not fully prevent viral replication [16,31], and other types of viral promoters also appear important to origin function. For example, there are no middle-mode promoters near *oriE*; instead this origin apparently depends on an early-mode promoter, which does not require viral transcription factors for activity [16]. Moreover, mutations that prevent late-mode viral transcription alter replication from T4 *oriC*, without affecting activity from the other origins (Brister, unpublished), raising the possibility that a late-mode promoter is required for activity from this origin.

Discontinuous lagging strand replication is normally primed by the T4-encoded gp61 primase [32-34]. Even though T4 primase is required only for lagging strand synthesis *in vitro*, the *in vivo *results are more complex. First, mutants deficient in primase show a severe DNA-delay phenotype, with very little DNA synthesis occurring early during infection [24,30,35,36]. This implies that primase activity contributes directly to early steps of T4 DNA replication. Either leading strand synthesis at some T4 origins is primed by primase, or normal viral replication requires the coupling of leading strand synthesis with primase-dependent lagging strand synthesis. Second, T4 DNA replication eventually reaches a remarkably vigorous level in primase-deficient infections, even when using a complete primase deletion mutant [24] (also see [37]). One published report suggested that the primase-independent replication was abolished by mutational inactivation of T4 endonuclease VII, leading to a model in which endonuclease VII cleavage of recombination intermediates provides primers for DNA synthesis [38]. However, repetition of this experiment revealed little or no decrease in endonuclease-deficient infections [39], and the strain used in the Mosig study was later found to contain an additional mutation that was contributing to the reduced replication (G. Mosig, personal communication to KNK). The mechanism of extensive DNA replication late in a primase-deficient infection remains unclear, but could possibly result from extensive priming by mRNA transcripts (perhaps in combination with endonuclease cleavage as suggested by Mosig [38]).

In other systems, there are examples of both primase- and transcript-mediated initiation of leading strand DNA synthesis from origins. A transcript is used to prime replication from the ColE1 plasmid origin, as well as mitochondrial DNA origins [40,41], yet primase is used to initiate replication from the major *E. coli *origin, *oriC *[42,43]. Indeed, there are even systems where both mechanisms of initiation are used within a single chromosome. For example, unlike *oriC*, R-loops are apparently used to initiate DNA synthesis at the *oriK *sites in *E. coli *(reviewed in [44]).

The molecular mechanism of T4 replication initiation has been investigated *in vitro *using R-loop substrates constructed by annealing an RNA oligonucleotide to supercoiled *oriF *plasmids [45]. Efficient replication of these preformed R-loop substrates does not require a promoter sequence, but a DUE is necessary. In fact, non-origin plasmids are efficiently replicated *in vitro *by the T4 replisome as long as they have a preformed R-loop within a DUE region, implying that the R-loop itself is the signal for replisome assembly on these substrates. Experiments using radioactively labeled R-loop RNA directly demonstrated that the RNA is used as the primer for DNA synthesis. Several viral proteins are required for significant replication of these R-loop substrates: DNA polymerase (gp43), polymerase clamp (gp45), clamp loader (gp44/62), and single-stranded DNA binding protein (gp32). In addition, without the replicative helicase (gp41), leading-strand synthesis is limited to a relatively short region (about 2.5 kb) and lagging strand synthesis is abolished. While gp41 can load without the helicase loading protein (gp59), the presence of gp59 greatly accelerates the process. Finally, replication on these covalently closed substrates is severely limited when the T4-encoded type II topoisomerase (gp39/52/60) is withheld, as expected due to the accumulation of positive supercoiling ahead of the fork.

Normal viral replication also requires gp59 protein, and though gene *59 *mutants make some DNA early, this synthesis is arrested as the infection progresses [5,46,47]. This deficiency was initially thought to reflect a unique requirement for gp59 in recombination-dependent replication (i.e., no requirement in origin-dependent replication). However, gp59 mutations also affect origin activity, reducing the total amount of origin-mediated DNA synthesis, mirroring the *in vitro *studies mentioned above [5]. Further defects are clearly visible at *oriG*, where gene *59 *mutations cause problems in the coupling of leading and lagging strand synthesis (but do not prevent replication initiation) [48].

The deleterious effects of gene *59 *mutations could reflect several biochemical activities that have been characterized *in vitro*. A major function of gp59 is loading of the replicative helicase gp41 [49]. Gp59 is a branch-specific DNA binding protein with a novel alpha-helical two-domain fold [50]. The gp59 protein is capable of binding a totally duplex fork, but requires a single-stranded gap of more than 5 nucleotides (on the arm corresponding to the lagging strand template) to load gp41 [51]. As expected from this loading activity, gp59 stimulates gp41 helicase activity on branched DNA substrates (e.g. Holliday junction-like molecules). Interestingly, gp59 has another function in the coordination of leading- and lagging-strand synthesis and in this context has been called a "gatekeeper". When gp59 binds to replication fork-like structures in the absence of gp41, it blocks extension by T4 DNA polymerase [45,48,52]. This inhibitory activity of gp59 presumably acts to prevent the generation of excessive single-stranded DNA and allow coordinated and coupled leading and lagging strand synthesis.

Unlike gp59, the viral gp41 helicase is required for extended replication of R-loop substrates *in vitro *(see above) and any appreciable replication during infection [15,45,53]. Yet, some viral replication is observed in gp59-deficient infections (see above), indicating that gp41 helicase can load onto origins at some rate through another means. T4 encodes at least two other helicases, UvsW and Dda, and earlier studies demonstrated that one of them, Dda, stimulates gp41-mediated replication *in vitro *[49]. It was therefore suggested that either gp59 or Dda was sufficient to load gp41 helicase at the T4 origins [49]. Consistent with this notion, *dda *mutants have a DNA delay phenotype and are deficient in early, presumably origin-mediated DNA synthesis, though replication rebounds at later times when it is dependent on viral recombination [15,46]. Moreover, *dda 59 *double mutants have a greater defect than either single mutant, essentially showing no replication (either early or late) and indicating a cumulative effect on origin activity [46].

Though there may be some functional overlap between Dda and gp59, DNA replication patterns indicate that each has distinct activities at the T4 origins [15]. Unlike *dda *mutations, which cause a generalized reduction in DNA synthesis that is particularly evident at *oriE*, gene *59 *mutations have little effect on replication from this origin [15]. This difference may indicate that *oriE *uses a different mechanism to initiate replication, one less dependent on gp59. This idea has been expressed before and may simply reflect the difference in sequence elements at *oriE *compared to the other origins. One protein in particular, RepEB, has also been implicated in *oriE *activity [16], but *repEB *mutations have a more generalized effect, reducing replication from all origins [15].

### Inactivation of origins at late times

The regulation of origin usage has been studied directly for *oriF *and *oriG*, the two origins known to function via an R-loop intermediate. One level of control is exerted by the change in the transcriptional program. The RNA within the *oriF *and *oriG *R-loops are initiated from MotA-dependent middle mode promoters, which are shut off as RNA polymerase is converted into the form for late transcription [28,29]. A second level of control is exerted when the UvsW helicase is expressed from its late promoter [54]. UvsW is a helicase with fairly broad specificity for various branched nucleic acids, including the R-loops that occur at *oriF *and *oriG *[55-57]. Thus, any existing R-loops at these origins are unwound when UvsW is synthesized. While not yet studied directly, R-loops may also occur at one or more other T4 origins (e.g. *oriE*), and thus the mechanisms of regulation could be identical to that of *oriF *and *oriG*. Further work is clearly needed to understand the regulation of other T4 origins.

As will be discussed in more detail below, mutational inactivation of T4 recombination proteins leads to the DNA arrest phenotype, characterized by a paucity of late DNA replication. The additional inactivation of UvsW suppresses this DNA arrest phenotype and allows high levels of DNA synthesis at late times [58-61]. The simplest explanation is that R-loop replication becomes dominant in these double-mutant infections at late times. If true, it seems likely that much of this late replication is initiated at R-loops formed at late promoters, but these "cryptic origin" locations have not yet been experimentally defined.

### Recombination-dependent replication

**Figure 2:**
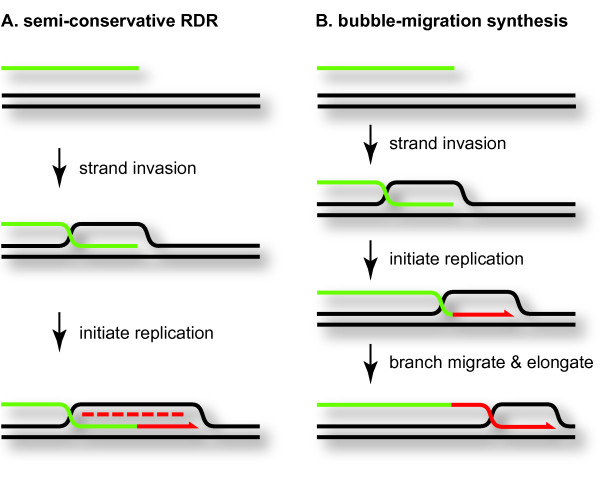
**Two modes of recombination-dependent replication (RDR) **. During semi-conservative RDR, primase action on the displaced strand of the D-loop allows lagging strand synthesis (panel A). In bubble-migration synthesis, lagging strand synthesis does not occur, and the newly synthesized single strand is extruded from the back of the D-loop as new DNA is synthesized at the front of the D-loop (panel B). In this and subsequent figures, new leading strand replication is in solid red and new lagging strand replication is in dashed red; the two starting molecules are differentiated by the green versus black colors.

The tight coupling of homologous genetic recombination and DNA replication was first recognized in the phage T4 system when it was found that mutational inactivation of recombination proteins leads to the DNA-arrest phenotype characterized by defective late replication [62]. Based on this and other data, Gisela Mosig proposed that genomic DNA replication can be initiated on the invading 3' ends of D-loop structures generated by the recombination machinery (Figure 2A) [63]. There is now abundant *in vivo *and *in vitro *evidence supporting this model for phage T4 DNA replication. T4 RDR is an important model for the linkage of recombination and replication, because it has become clear that recombination provides a backup method for restarting DNA replication in both prokaryotes and eukaryotes (see below).

### RDR on the phage genome

The infecting T4 DNA is a linear molecule, and early genetic results showed that the (randomly located) DNA ends are preferential sites for homologous genetic recombination [64-66]. When an origin-initiated replication fork reaches one of the DNA ends, one of the two daughter molecules should contain a single-stranded 3' end that is competent for strand invasion and D-loop formation; the other daughter molecule is also presumably competent for strand invasion after processing to generate a 3' end. The complementary sequence that is invaded could be at the other end of the same DNA molecule, since the infecting T4 DNA is terminally redundant, or it may be within the interior region of a co-infecting T4 DNA molecule, since T4 DNA is also circularly permuted. In this way, the process of RDR can in principle initiate soon after an origin-initiated fork reaches a genomic end. As will be described below, RDR or some variant thereof might be needed to continue replication well before origin-initiated forks reach the genome ends. The overall role of RDR in genome replication and the relationship of RDR to the eventual packaging of phage DNA are discussed in detail elsewhere [6,67].

RDR of the phage genome is abolished or greatly reduced by mutational inactivation of most T4-encoded recombination proteins (see [68] for review on the biochemistry of T4 recombination proteins). The strongest DNA arrest phenotypes are caused by inactivation of gp46/47 or gp59, and correspondingly, these are essential proteins. Inactivation of the non-essential UvsX and UvsY proteins eliminate most but not all late DNA replication. These two proteins catalyze the strand invasion reaction that generates D-loops, and so one might expect RDR to be totally abolished. However, a significant amount of T4 genetic recombination still occurs in the absence of UvsX or UvsY, and this has been ascribed to a single-strand annealing pathway [69,70]. Single-strand annealing intermediates may also be used to initiate RDR, which could explain the residual late DNA replication in UvsX or UvsY knockout mutants.

The *uvsW *gene is in the same recombinational repair pathway as *uvsX *and *uvsY *[71]. However, the *uvsW *gene product was not originally implicated in the process of RDR because *uvsW *knockout mutations do not block late DNA replication [71]. This inference was probably misleading - as described above, the UvsW helicase apparently unwinds R-loops that could otherwise trigger replication at late times. Thus, inactivation of UvsW could simultaneously reduce or eliminate RDR and activate an R-loop dependent mechanism of late replication, resulting in no net decrease in late DNA replication [54]. Consistent with this model, a *uvsW *mutant has reduced recombination and was shown to be defective in generating phage DNA longer than unit length (in alkaline sucrose gradients) [71]. In addition, UvsW is required for a plasmid-based model for RDR [55] (see below).

The one T4 recombination function that is not required for RDR is endonuclease VII, which resolves Holliday junctions and other branched DNA structures [72,73]. The major function of endonuclease VII during infection is to resolve DNA branches during DNA packaging [74,75]. Because this is a very late step in genetic recombination, the lack of a role in RDR is unsurprising.

### Plasmid model systems for RDR

Plasmid model systems have been productive for analyzing the mechanism of RDR *in vivo*, and have revealed a very close relationship between repair of DSBs and the process of RDR. Plasmids with homology to the T4 genome but no T4 replication origin are replicated during a phage T4 infection, as long as T4-induced host DNA breakdown is prevented [76-78]. This plasmid replication is not dependent on particular T4 sequences, because even plasmid pBR322 can be replicated when the infecting T4 carries an integrated copy of the plasmid [76]. Plasmid replication requires T4 recombination proteins, arguing that it occurs by RDR [77]. The products of plasmid replication in a T4 infection consist mostly of long plasmid concatamers, arguing that rolling circle replication is induced, but the mechanism of rolling circle formation is unknown [79].

The remarkable discovery of mobile group I introns in T4 [80] led to a simple way to introduce site-specific DSBs during a T4 infection, which has been valuable for *in vivo *studies of T4 RDR. These introns encode site-specific DNA endonucleases, such as the endonuclease I-*Tev*I from the intron of the T4 *td *gene (see below for discussion of the intron mobility/DSB repair events; also see [81]). The recognition site for I-*Tev*I (or another intron-encoded nuclease, SegC) has been introduced into recombinant plasmids and also into ectopic locations in the T4 genome, and in either case, the site is cleaved efficiently during a normal T4 infection when the endonuclease is expressed [76,82-86]. If the regions adjacent to the cut site have a homologous DNA target, either in the T4 genome or another segment of a plasmid residing in the same cell, coupled recombination/replication reactions are efficiently induced [76,79,87].

Using such model systems for RDR, it was shown that T4 recombination proteins UvsX, UvsY, UvsW, gp46/47, and gp59 are required for extensive DSB-directed replication, as are the expected T4 replication fork proteins (gp43, gp44/62, gp45, gp32, gp41, gp61; delayed replication of the plasmid occurs in the gp61-deficient infection, similar to the delayed replication of chromosomal DNA) [24,55,77]. In addition, by limiting the homology to just one side of the break, a single double-strand end was shown to be sufficient to induce RDR, as predicted by the Mosig model [76,86].

### Molecular mechanism of RDR

The heart of the RDR process is the strand-invasion reaction that creates D-loops, which is described in more detail in the review on T4 recombination [68]. Briefly, DNA ends are prepared for strand invasion by the gp46/47 helicase/nuclease complex, transient regions of ssDNA are coated by the single-strand binding protein gp32, UvsY acts as a mediator protein in loading UvsX onto gp32-coated ssDNA, and UvsX is the strand-invasion protein (RecA and Rad51 homolog). Recent evidence argues that the UvsW helicase also plays a direct role in strand invasion, promoting 3-strand branch migration to stabilize the D-loop [88].

As described in more detail by Kreuzer and Morrical [6], early reconstitution of a T4 RDR reaction *in vitro *generated a conservative replication reaction called bubble-migration synthesis [89]. In bubble-migration synthesis, the 3' invading end in the D-loop is extended by DNA polymerase as the junction at the back of the D-loop undergoes branch migration in the same direction (Figure 2B). The net result is that a newly synthesized single-strand copy is created and then quickly extruded from its template, and lagging-strand synthesis does not occur within the D-loop.

In the RDR reactions analyzed by Formosa and Alberts [90], the T4 DNA polymerase holoenzyme complex (polymerase gp43, clamp gp45 and clamp loader gp44/62) catalyzed synthesis in reactions containing only UvsX and gp32. Interestingly, synthesis did not occur if the host RecA protein was substituted for UvsX (even if host SSB protein was added), suggesting that the T4 polymerase complex has specific interactions with the phage-encoded strand-exchange protein. The extent of synthesis was limited unless a helicase was added to facilitate parental DNA unwinding - Dda was used in these initial experiments and allowed extensive bubble-migration synthesis [90].

Since the publication of Molecular Biology of Bacteriophage T4 in 1994 [91], much progress has been made in understanding the mechanism of loading of the helicase/primase complex onto D-loops. When T4 RDR reactions are supplemented with gp59, gp41 and gp61, lagging-strand synthesis is efficiently reconstituted on the displaced strand of the D-loop, and a conventional semi-conservative replication fork is established (Figure 2A) (see [6]). As described above, gp59 is a branch-specific DNA binding protein that loads gp41, and gp59 interacts specifically with both gp41 and gp32 in the loading reaction [50,51,92-97]. Jones et al. [94] showed that gp59 can load helicase onto a structure that closely resembles a D-loop, reflecting its role in RDR. Once the replicative helicase is loaded onto the displaced strand of the D-loop (which becomes the lagging-strand template), leading strand synthesis by T4 DNA polymerase (gp43) is activated. Because the T4 primase gp61 binds to and functions with gp41 (see [7]), loading of gp41 is critical to begin lagging-strand synthesis as well.

### Overlap between origin- and recombination-dependent mechanisms

The transition between origin- and recombination-dependent replication is not entirely clear cut during T4 infection, and there is significant interplay between the two replication modes. Moreover, the relationship between origin- and recombination-dependent replication is dynamic, which is clearly seen in experiments with varying multiplicities of infection. In singly infected cells, there is a prolonged period early during infection when the recombination protein UvsX is not required for replication. Yet, when cells are infected with an average of five viruses, the timing changes, and even very early replication is dependent on UvsX [5]. Though the mechanism of this regulation is not clear, it is evident that the infection program can somehow sense the amount of infecting viral DNA and switch replication modes under conditions where there are ample templates for RDR.

Recombination proteins also appear to be more important to replication from some origins compared to others. As mentioned earlier, genetic requirements vary among the multiple T4 replication origins that are active within a single population of infected cells. At least one origin, *oriA*, appears more active later during infection, when replication is dependent on the viral recombination machinery. Moreover, replication from this origin is significantly reduced when the viral recombination protein UvsX is mutated [5]. Though these observations underscore a role for T4 recombination machinery at *oriA*, it is not clear whether RDR is preferentially initiated near *oriA *or if normal *oriA*-mediated replication is partially dependent on UvsX.

One hint to the role of UvsX during origin-mediated replication comes from the apparently slow movement of replication forks across the T4 chromosome. Once initiated, T4 replication forks do not simply progress from an origin to the ends of the chromosome at the 30-45 kb per minute rate observed *in vitro *[5]. Rather, replication forks appear to move more slowly than expected, resulting in the accumulation of sub-genomic length DNAs early during infection. Only later are these short DNAs efficiently elongated into full-length genomes. This behavior was initially noticed by Cunningham and Berger [58], who analyzed the length of newly replicated single-stranded DNA using alkaline sucrose gradients. They also showed that efficient maturation of nascent DNAs into full genome length products requires the viral replication proteins UvsX or UvsY. A similar effect was observed during array studies where the elongation of nascent DNAs was greatly delayed in *uvsX *mutant infections compared to normal infections [5].

So why is there a delay in the elongation of T4 nascent DNAs? One possibility is that physical factors (e.g. tightly bound proteins) impede the progress of the replication forks across the T4 chromosome, causing replisomes to stall or disassociate from the DNA template. Rescue of model stalled forks *in vitro *can be catalyzed by UvsX and either gp41 helicase (with gp59) or Dda helicase [98]. Thus, one model is that UvsX is required *in vivo *to restart origin-initiated forks that have stalled before completing replication, and so the elongation of nascent DNAs is compromised during *uvsX *mutant infections.

Several factors may impede the progress of replication forks (also see below). T4 replication occurs concurrently with transcription during infection [19] (Brister, unpublished results), so replisomes must compete with the transcriptional machinery for template. Head-on collisions with RNA polymerase cause pausing of T4 replisomes *in vitro *[99], and undulating patterns of T4 transcription imply that replication forks must eventually pass through regions of head-on transcription. Furthermore, if multiple origins are active on a single chromosome, then replication forks initiated at different origins would speed towards one another, plowing through the duplex template. In this scenario intervening sequences would be wound into impassable torsion springs, and T4 topoisomerase (gp39/52/60) would be necessary to relax the duplex and allow progression. Indeed, gene *52 *mutants produce shorter than normal DNA replication products early during infection, similar to *uvsX *mutants [100].

### Interrelationship between replication, recombination and repair

Studies in many different biological systems have uncovered key roles of recombination proteins in the replication of damaged DNA [1-4]. One major set of pathways involves the repair of DSBs and broken replication forks. In addition, recombination proteins are involved in multiple pathways proposed for replication fork restart after blockage by non-coding lesions, some pathways coupled to repair of the DNA damage and others that result in bypass of the damage. Here, we briefly review unique contributions to this field that emerged from the phage T4 system.

### Tight linkage of DSB repair and RDR

As indicated above, DSB repair in phage T4 is closely related to the process of RDR. Studies of DSB repair were greatly accelerated by the discovery of the mobile group I introns and their associated endonucleases. Intron mobility involves the generation of a DSB within the recipient (initially intron-free) DNA by an intron endonuclease, followed by a DSB repair reaction that introduces a copy of the intron from the donor DNA, such that both recipient and donor end up with a copy of the intron [80,81,101].

**Figure 3:**
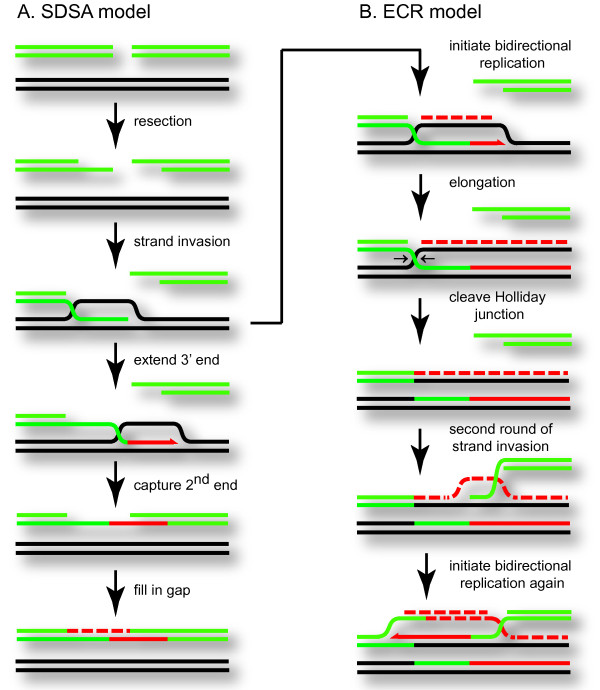
**Double-strand break repair models **. The SDSA model for DSB repair invokes a limited amount of bubble-migration synthesis using one end of a double-strand break, followed by extrusion of the extended 3' end and capture of the second broken end (panel A). The extensive chromosomal replication (ECR) model invokes two successive rounds of semi-conservative replication (panel B). Depending on which product of the first round of replication is chosen for the second round of strand invasion, the two broken ends of the original double-strand break can end up in different molecules rather than being linked back together again. The final stages of elongation are not shown, but would result in three complete product molecules.

A variety of approaches have been used to study the detailed mechanism of DSB repair *in vivo *using intron endonuclease-mediated DSBs. One series of studies using a plasmid model system indicated that the DSBs are repaired by a pathway called synthesis-dependent strand annealing (SDSA), in which the induced DNA replication is limited to the region near the DSB (Figure 3A) [102,103]. The SDSA repair mechanism is closely related to the bubble-migration reaction described above, and has been implicated in DSB repair in eukaryotic systems such as Drosophila [104,105]. Other studies, however, argue that the DSB leads to the generation of fully functional replication forks in a process that is very closely related to the RDR pathway that occurs in the phage genome [79,85,87,106]. This so-called extensive chromosomal replication (ECR) model leads to *bona fide *DSB repair, even though the two broken ends of the DSB can end up in different molecules (Figure 3B).

A major difference between the SDSA and ECR models for DSB repair is that SDSA does not involve primase (gp61)-dependent lagging strand synthesis, while the ECR model does. Perhaps either repair model can occur when a DSB occurs on the phage genome, but the choice of pathways depends on whether the helicase/primase complex is successfully loaded onto the displaced strand of the initial D-loop. Considering that gp59 efficiently inhibits polymerase and loads helicase/primase (see above), it is difficult to see how the bubble-migration pathway and SDSA could occur *in vivo*, unless there is some additional level of regulation that has not yet been uncovered. Shcherbakov et al. [106] have presented additional evidence that DSBs trigger normal replication like that postulated in the ECR model, and provided arguments against a major role for the SDSA pathway during wild-type T4 infections.

If a DNA end can trigger a new replication fork by invading homologous DNA, there would seem to be no need to coordinate the processing of the two ends of a DSB - each could simply start a new replication fork on any homologous DNA molecule. Indeed, if the two DNA segments flanking a DSB are homologous to two different plasmid molecules, the DSB is repaired by inducing replication of both plasmids [86]. While this result clearly shows that the two ends can act independently when forced to do so, other experiments demonstrate that the two broken ends of a DSB are often repaired in a coordinated fashion, using the same template molecule [86,106]. Moreover, Shcherbakov et al. [106] presented striking evidence that the end coordination is dependent on the gp46/47 complex. The eukaryotic homolog, Rad50/Mre11, has also been implicated in end coordination in DSB repair by a mechanism involving tethering of the two ends via a protein bridge [107,108]. How does end tethering relate to the extensive replication triggered by the broken ends? The simplest explanation is that one end of the DSB triggers a new replication fork on a homolog, and then the second broken end invades one of the two newly-replicated products from that first replication event and triggers a second replication fork in the opposite direction, as diagrammed in Figure 3B[86,106].

### Replication fork blockage and restart

Replication forks can be blocked or stalled by template lesions, lack of nucleotide substrates, or problems with the replication apparatus. In addition to the natural blockage that appears to occur in normal infections (see above), the consequences of fork blockage and possible pathways for fork restart have been studied using two different inhibitors. First, hydroxyurea (HU) inhibits the reduction of ribonucleotides to deoxyribonucleotides and thereby depletes the nucleotide precursors for replication [109]. Second, the topoisomerase inhibitor 4'-(9-acridinylamino)-methanesulfon-*m*-anisidide (*m*-AMSA) stabilizes covalent topoisomerase-DNA complexes and thereby physically blocks T4 replication forks [110].

Wild-type T4 induces breakdown of host DNA, providing a significant source of deoxynucleotide precursors for phage replication and thereby making the phage relatively resistant to HU. One class of HU hypersensitive mutants consists of those defective in the breakdown of host DNA (e.g., *denA *which encodes DNA endonuclease II) [111,112]. A second well-studied HU hypersensitive mutant class consists of those with knockouts of the *uvsW *gene [71,113]. These mutants are not defective in host DNA breakdown, and the HU hypersensitivity of *uvsW *mutants was shown to result from a different genetic pathway than that of *denA *mutants. We will suggest below that the UvsW protein plays a special role in processing blocked replication forks, namely that it catalyzes a process called replication fork regression. We also suggest that fork regression might somehow lead to efficient replication fork restart, although the details are unclear. Interestingly, the HU hypersensitivity of *uvsW *knockout mutants can be eliminated by additional knockout of *uvsX *or *uvsY *[58]. This result suggests that the UvsXY homologous recombination system creates some kind of toxic intermediate/product from stalled replication forks when the UvsW protein is unavailable - the nature of this toxic structure is currently unknown.

The phage T4 type II DNA topoisomerase is sensitive to anticancer agents, including *m*-AMSA, that inhibit mammalian type II topoisomerases [114]. For both enzymes, the drugs stabilize an otherwise transient intermediate in which the enzyme is covalently attached to DNA with a latent enzyme-induced DNA break at the site of linkage. Treatment of phage T4 infections with *m*-AMSA thereby leads to replication fork blockage at the sites of topoisomerase action [110]. Interestingly, the blocked replication fork does not immediately resume synthesis when the topoisomerase dissociates from its site of action (and reseals the latent DNA break in the process). This result strongly suggests that key components of the replisome had been disassembled upon fork blockage, so that a fork restart pathway must be used to resume DNA replication.

Mutations in genes *46/47*, *59*, *uvsX*, *uvsY*, and *uvsW *each lead to hypersensitivity to *m*-AMSA, arguing that the RDR pathway or some close variant is required to survive damage caused by *m*-AMSA [115,116]. Consistent with this model, continued replication of an origin-containing plasmid in the presence of the drug (but not in its absence) was shown to be inhibited in a *46 uvsX *double knockout mutant [110]. The simplest interpretation is that the T4 RDR system allows the restart of replication after the fork blockage event. One plausible scenario is that the blocked replication forks are especially prone to cleavage, for example by a recombination nuclease such as endonuclease VII (gp49), and that the RDR pathway provides a mechanism to restart the broken forks. Evidence supporting this view was obtained when it was found that endonuclease VII can indeed cleave blocked replication forks *in vitro*, and that blocked forks accumulate to a higher level during infections with a gene *49 *knockout mutant [117]. These latter results led the authors to propose a "collateral damage" model, in which cytotoxic DNA damage from these anticancer agents results from endonuclease-mediated cleavage of stalled replication forks. It should be noted that the processing of forks stalled by HU and *m*-AMSA must differ significantly, because inactivation of *uvsX *or *uvsY *causes hypersensitivity to *m*-AMSA but not to HU, suggesting that only *m*-AMSA leads to high levels of broken forks.

**Figure 4:**
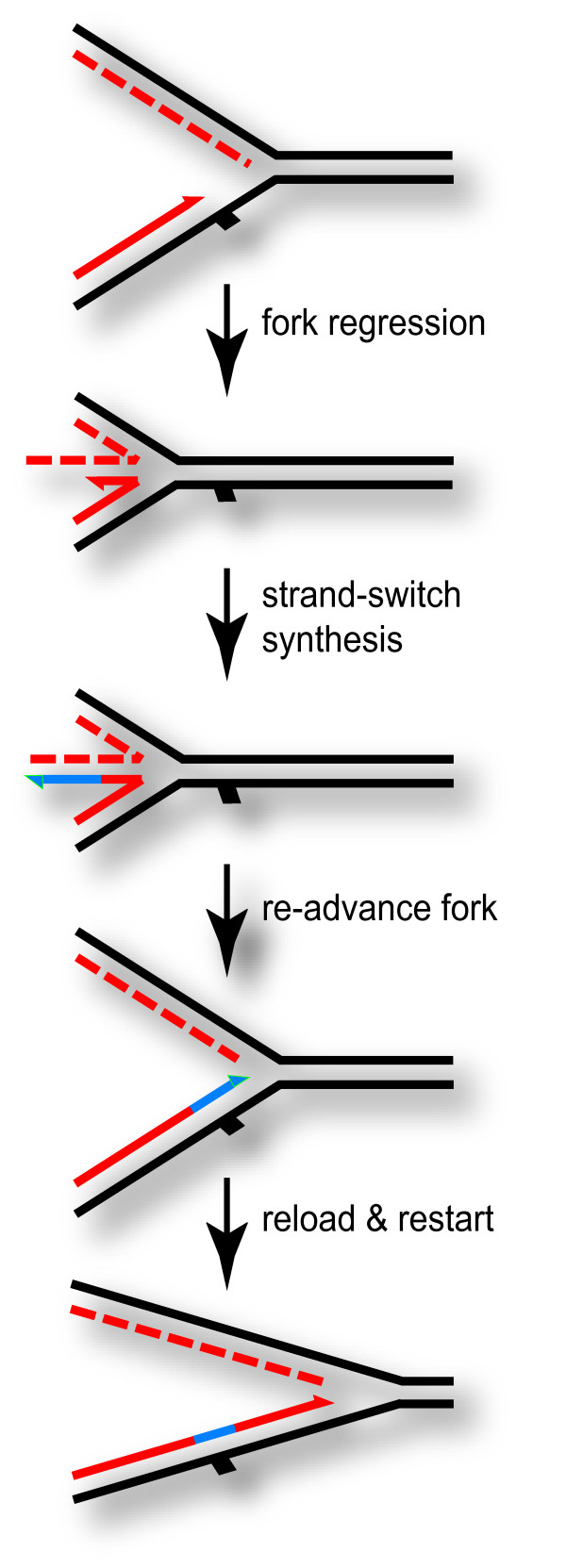
**Fork regression model for bypass of leading-strand damage **. In this model, leading-strand replication encounters a blocking lesion (solid rectangle) while lagging-strand replication proceeds past the site of the lesion. Replication fork regression leads to an accurate template for extension of the blocked leading-strand product, and re-advancing the fork (reversing the regression) puts the newly synthesized segment of leading-strand product (blue line) opposite the blocking lesion.

In an attempt to further study the restart pathway(s) of blocked replication forks in T4 infections, Long and Kreuzer [118,119] analyzed the fork-shaped intermediates ("origin forks") that accumulate at *oriG *after one replication fork has left the origin region. Novel intermediates were detected by two-dimensional gel electrophoresis at a relatively low abundance in wild-type infections, and these were ascribed to replication fork regression [118]. Replication fork regression is a process in which the two newly synthesized strands of a replication fork are unwound from their complementary partners and rewound together, backing up (regressing) the location of the fork along the DNA. Many years ago, Higgins et al. [120] proposed this general model as a step in the accurate replication of damaged DNA in mammalian cells (Figure 4).

What is the significance of the fork regression at *oriG*? A clue was uncovered when the amount of regressed fork was found to be substantially increased when either gp46/47 or gp49 (endonuclease VII) was mutationally inactivated [118]. The authors therefore proposed that gp46/47 normally processes the extruded duplex of the regressed fork, and that endonuclease VII can cleave the regressed fork (which resembles a Holliday junction). Either of these steps could initiate a fork restart pathway, and it is possible that they normally function together as a single fork reactivation pathway (see [118]). One possible model for the fork restart pathway is that the extruded duplex in the regressed fork undergoes a strand invasion reaction ahead of the position of the fork, and thereby initiates replication by an RDR reaction (also see above).

In a subsequent study, the UvsW helicase was shown to be required for detection of the regressed forks *in vivo*, and also that purified UvsW helicase can catalyze fork regression *in vitro *with forked DNA isolated from a T4 infection [119]. These results strongly suggest that fork regression is an active process that contributes to survival after DNA damage, since *uvsW *knockout mutants are hypersensitive to DNA damaging agents [71,113,116].

Is fork regression required for restart of stalled forks (or other fork-shaped structures) in a T4 infection? The above experiments do not provide a clear answer to this question, because the regressed fork intermediates were only a relatively small subset of the blocked forks and because some of the RDR proteins could themselves be involved in loading a replisome onto a simple fork structure. Indeed, gp59 can bind to simple fork structures *in vitro *and load the gp41 helicase (see above), supporting the possibility of a simple direct loading pathway. Perhaps multiple processing pathways compete for access to blocked/stalled forks in T4, and the different pathways have unique capabilities to resolve different kinds of problems (e.g., different forms of DNA damage).

### Replication of damaged DNA by strand switching

The general involvement of recombination in the successful replication of damaged DNA was first uncovered in the pioneering experiments of Luria [121], who discovered the phenomenon of multiplicity reactivation (MR). In MR, co-infection with multiple phages, each of which has extensive DNA damage, results in viable progeny, while single infections with the same phage particles result in no burst. Subsequent studies clearly showed the involvement of both replication and recombination functions in MR (reviewed by [122,123]). A favored model to explain MR involves DNA polymerase strand switching upon encounter with DNA damage, but the molecular details of MR have yet to be elucidated (see [123] for discussion of this and other models).

While further experiments are needed to test the strand-switching model for MR, studies over the last 15 years have provided direct evidence for strand switching in the T4 system. Strand switching may also play important roles in the process of post-replication recombination repair (PRRR) and a pathway called replication repair (see [123]).

Strand switching events can promote the accurate replication of damaged DNA when the second template is a *bona fide *homolog, either from the opposite daughter duplex behind a replication fork or from another homologous DNA molecule. An *in vitro *model for this process was established by Kadyrov and Drake [98,124], who engineered replication-fork like substrates with a blocking lesion in the leading strand and a pre-existing lagging strand product that extended past the site of blockage. They were able to demonstrate that the leading-strand product can be extended past the site of blockage by a strand switching event that allows extension using the longer lagging-strand product as template. The simplest way to model the strand switching event is by replication fork regression, followed by polymerase extension on the extruded duplex [98] (Figure 4). To complete the error-free bypass of the DNA damage, a second strand switching event is needed, and this can occur by reversal of the fork regression process. This event was also detected in the studies of Kadyrov and Drake [98].

The *in vitro *strand switching analyzed by Kadyrov and Drake had several properties that resemble the replication of damaged DNA during T4 infections. Certain alleles of gene *32 *and *41 *compromise a process called replication repair *in vivo*, and these same alleles greatly reduced the strand switching process *in vitro *[124]. Furthermore, the UvsX recombinase is centrally important in survival after DNA damage *in vivo*, and greatly stimulated the strand switching reaction *in vitro *[98]. The Dda helicase was also shown to stimulate the *in vitro *strand switching, but Kadyrov and Drake [98] suggested that the UvsW helicase was more likely to promote this role *in vivo *based on the phenotypes of *dda *and *uvsW *mutants. Consistent with this suggestion, the UvsW protein was subsequently shown to catalyze fork regression (see above), and very recent evidence has directly demonstrated *in vitro *strand switching promoted by UvsW [125].

Even earlier evidence for strand switching *in vitro *came from reactions in which the polymerase changed templates, presumably at inverted repeat sequences [37,126]. In this reaction, the 3' end of a newly replicated strand base pairs with a short complementary sequence that happens to be on the same strand, resulting in a replication event in which the same strand is used as both template and primer (also see [127]). This reaction is genetically aberrant and would create genome rearrangements rather than assist in the replication of damaged DNA. Indeed, Schultz et al. [128] presented evidence that a similar kind of aberrant strand switching can lead to "templated" mutations during a T4 infection. These mutations apparently arise from sequential strand switching events in which DNA polymerase copies an imperfect repeat elsewhere in the template and then returns to the correct initial location on the template. Interestingly, these templated mutations became more frequent with certain mutations in genes *32*, *41 *and *uvsX*, arguing that these proteins normally help to accurately direct the template switching events, e.g. to the opposite daughter strand rather than to ectopic locations elsewhere in the genome.

## Conclusions and perspectives

Phage T4 has continued to provide an important model system for studies of the mechanisms of DNA replication, recombination and repair, and in several cases has led the way in illuminating the interconnections between these processes. A major example is RDR, a process that was first studied in detail in phage T4, that was originally thought to be an odd peculiarity of the phage's life cycle, but that is now appreciated as central in the completion of cellular genomic replication and the repair of DSB's in prokaryotic and eukaryotic chromosomes (for reviews, see [1-4]). Recombination-related pathways, including RDR, strand-switching and replication fork regression, are now appreciated to be critical in the maintenance of genome stability in mammalian systems and thereby important in cancer biology. There seems to be particularly strong parallels between DNA metabolism in phage T4 and in eukaryotic mitochondrial DNA and mitochondrial plasmid DNA. In these systems, evidence has been obtained for both R-loop-mediated replication and RDR, as well as EM data showing branched concatameric DNA similar to that of intracellular replicating T4 DNA [129-135].

While we have learned much about how T4 initiates replication at both origins and from recombination structures, many important questions remain to be answered. We will close with a few of the most interesting, which suggest that important principles and lessons remain to be uncovered using the T4 model system:

(i) What are the rules governing R-loop formation at *oriF *and *oriG*?

(ii) Do other T4 origins use an R-loop mechanism or some other initiation process?

(iii) What are the factors that govern origin usage and change the pattern of origin function?

(iv) What are the precise roles of recombination proteins, gp59 and Dda in origin usage?

(v) Why do replication forks often fail to complete replication, or move very slowly, at early times of infection?

(vi) Does T4 use a "direct restart" pathway *in vivo*, in which the replisome is loaded directly onto a fork structure?

(vii) What are the detailed roles of the gp46/47 complex, the homolog of eukaryotic Mre11/Rad50 complex?

(viii) How does replication fork regression contribute to replication fork restart?

(ix) How frequently does T4 use strand switching mechanisms *in vivo*, which proteins are required, and how is the process regulated?

## Competing interests

The authors declare that they have no competing interests.

## Authors' contributions

KK wrote the first drafts of the "Introduction", the section on "Recombination-dependent replication", the section on "Interrelationship between replication, recombination and repair", and the "Conclusions and perspectives"; JRB wrote the first draft of the section on "Overlap between origin- and recombination-dependent mechanisms"; both authors contributed to the first draft of the section on "Origin-dependent replication"; both authors revised all sections and read and approved the final draft.
